# The Society for the State Registration of Trained Nurses

**Published:** 1913-08-09

**Authors:** E. W. Morris

**Affiliations:** Secretary. London Hospital, Whitechapel, E.


### The Society for the State Registration of Trained Nurses.

*To the Editor of* The Hospital.

Sir,—I am desired to lay before you the following correspondence which has passed between the Society for the State Registration of Trained Nurses and the Chairman of the London Hospital.—Yours very truly,

E. W. Morris, Secretary.

London Hospital, Whitechapel, E.,
July 31, 1913.

[Copy.]

The Society for the State Registration of Trained Nurses,

